# The role of melatonin on miRNAs modulation in triple-negative breast cancer cells

**DOI:** 10.1371/journal.pone.0228062

**Published:** 2020-02-03

**Authors:** Lívia C. Ferreira, Francesca Orso, Daniela Dettori, Jéssica Z. Lacerda, Thaiz F. Borin, Daniela Taverna, Debora A. P. C. Zuccari

**Affiliations:** 1 Department of Biology, Universidade Estadual Paulista "Júlio de Mesquita Filho", São José do Rio Preto, São Paulo, Brazil; 2 Molecular Biotechnology Center (MBC), Department Molecular Biotechnology and Health Sciences, University of Torino, Torino, Italy; 3 Department of Biochemistry and Molecular Biology, Tumor Angiogenesis Laboratory, Augusta University, Augusta, Georgia, United States of America; 4 Department of Molecular Biology, Faculdade de Medicina de São José do Rio Preto, São José do Rio Preto, São Paulo, Brazil; Beijing Cancer Hospital, CHINA

## Abstract

Melatonin, a hormone secreted by pineal gland, exerts antimetastatic effects by reducing tumor cell proliferation, migration and invasion. MicroRNAs (miRNAs) are small, non-coding RNAs that play a crucial role in regulation of gene expression and biological processes of the cells. Herein, we search for a link between the tumor/metastatic-suppressive actions of melatonin and miRNA expression in triple-negative breast cancer cells. We demonstrated that melatonin exerts its anti-tumor actions by reducing proliferation, migration and c-Myc expression of triple negative breast cancer cells. By using Taqman-based assays, we analyzed the expression levels of a set of miRNAs following melatonin treatment of triple negative breast cancer cells and we identified 17 differentially expressed miRNAs, 6 down-regulated and 11 up-regulated. We focused on the anti-metastatic miR-148b and the oncogenic miR-210 both up-regulated by melatonin treatment and studied the effect of their modulation on melatonin-mediated impairment of tumor progression. Surprisingly, when miR-148b or miR-210 were depleted in triple-negative breast cancer cells, using a specific miR-148b sponge or anti-miR-210, melatonin effects on migration inhibition and c-myc downregulation were still visible suggesting that the increase of miR-148b and miR-210 expression observed following melatonin treatment was not required for the efficacy of melatonin action. Nevertheless, ours results suggest that melatonin exhibit a compound for metastatic trait inhibition, especially in MDA-MB-231 breast cancer cells even if a direct link between modulation of expression of certain proteins or miRNAs and melatonin effects has still to be established.

## Introduction

Breast cancer is the most common type of cancer, and the second major cause of death in women worldwide [1]. The high mortality rate due to this neoplasm is intrinsically related to the occurrence of metastasis, which affects more than 90% of the patients. Despite the high incidence, the early diagnosis and the introduction of more effective treatments have allowed the decrease in deaths and have improved the quality of life of patients with the disease [2]. The progression of breast cancer depends on the ability of cells to invade and colonize distant sites [3]. Dissemination of tumor cells is a complex multi-step process, including detachment of primary tumor cells, invasion of the local tumor microenvironment, survival in the circulation and extravasation in other tissues [4].

Metastasis formation occurs through several mechanisms, and currently microRNAs (miRNAs) have been investigated as possible determinants of this process [5]. miRNAs are small molecules of RNA, with about 20–22 nucleotides, regulating gene expression at the post-transcriptional level, and are able to induce gene silencing after pairing with target molecules of messenger RNA (mRNA), leading to a destabilization or degradation of these targets. One miRNA could bind to hundreds of different mRNAs, regulating various cellular processes [6,7]. The literature reports the involvement of miRNAs in tumor suppression [8] and oncogenesis [9]. Therefore, miRNA deregulation plays an important role in proliferation, invasion, differentiation, apoptosis and cell resistance in various types of cancer [10]. Because of the complexity of miRNA involvement in the formation of breast cancer and the metastasis process, it becomes essential to investigate miRNA functions for advanced therapeutic strategies.

Melatonin, the principal hormone produced and secreted by the pineal gland, is effective in reducing the growth and development of several tumors, in particular estrogen-dependent breast cancer[11,12]. Furthermore, it has modulatory oncostatic effects on the cytoskeleton and it is able to inhibit the invasiveness of tumor cells [13–15]. A recent study of Mao et al. revealed that melatonin seems to be involved in the suppression of Epithelial to Mesenchymal Transition (EMT), either by promoting Mesenchymal-to-Epithelial Transition (MET), and/or by inhibiting key signaling pathways associated with the later stages of metastasis[16]. Currently, there is extensive knowledge of the intracellular signaling pathways of melatonin. However, its ability to modulate intracellular processes is extremely complex and still requires further studies [13,17,18].

Lee et al. made the first study of the effect of melatonin on miRNA modulation on the non-metastatic breast cancer cell line MCF-7. The results demonstrated that physiological levels of melatonin can modulate the expression of miRNAs, promoting an antiproliferative action in breast cancer [19]. Recently, Sohn et al. demonstrated the action of melatonin in increasing expression of miR-3195 and miR-374b in prostate tumor cells [20]. Here, the high expression of these molecules led to decreased levels of genes related to angiogenesis and metastasis, such as hypoxia-inducible factors (HIF-1α) and VEGF. Considering that only a few studies of miRNA regulation by melatonin exist so far, it remains essential to understand miRNA expression, and through which pathways melatonin can regulate these small molecules. In this study, we investigate the association between the metastatic-suppressive actions of melatonin and miRNA functions in the MDA-MB-231 triple-negative breast cancer cell line. Based on the findings, we suggest that melatonin regulates the metastatic abilities of MDA-MB-231 triple-negative breast cancer cells and leads to miRNA modulations. However, anti-metastatic actions of melatonin are not directly related to specific miRNA modulations.

## Methods

### Cell culture

Three different breast tumor lines were used: MDA-MB-231 (metastatic and triple-negative), 4175-TGL (metastatic derived from MDA-MB-231 cell line) and MCF-7 (non-metastatic and triple-positive). The breast tumor cells were cultured in DMEM medium supplemented with 10% FBS (Biochrom AG, Berlin, Germany), 1 mM pyruvate sodium, 25 mM HEPES (pH 7.4), and 100 mg/ml Gentamicin (all from Gibco, Invitrogen Life Technologies, Carlsbad, CA, USA).

### Proliferation assay

Cells were seeded at 5 × 10^3^ cells / in 96 well plates and re-suspended in a medium supplemented with 2% FBS. After 24 h, the cells received treatments with melatonin (100 nM and 1mM) and were allowed to grow for 24, 48, 72, 96 and 120 h. After treatment, the cells were fixed with 2.5% glutaraldehyde and stained with 0.2% crystal violet. The dye was solubilized using 10% acetic acid and the samples were homogenized and absorbance at 570 nm read using the Glomax Multi-Detection System (Promega).

### Migration assay

A migration assay was performed to evaluate the capability of the cell to migrate after treatment with melatonin (1 mM). For the experiment, we used 24-well plates including inserts (transwell^®^) having pores approximately 8 μm in diameter (Costar, Corning Incorporated). Tumor cells were plated at 1 x 10^5^ and re-suspended in 500 μl of serum-free medium in the upper compartment of the insert, while 600 μl of medium supplemented with 20% FBS was added in the lower compartment. The cells were treated with melatonin for a total of 48 h and incubated in a CO2 chamber at 37 °C overnight. Then the remaining in the lower compartment fixed in 2.5% glutaraldehyde for 20 minutes and stained with 0.2% violet crystal for 20 minutes at room temperature. Finally, all remaining cells in the lower part of the insert were counted using Zeiss Axiovert 200M microscope.

### Generation of stable modified cell lines and transient transfections

miR-148b depleted 4175-TGL expressed a specific miR-148bsponge with sequences designed to contain eight miRNA binding sites interrupted by 15-nts spacers to be perfectly complementary to the miR-148b seed region, with a bulge position 9–12 to prevent undesired cleavage of the sponge RNA as previously described in Penna et al [21]. Briefly, sponges were synthesized by DNA 2.0, cloned into pJ241 plasmids, excised using flanking HindIII sites, blunted and subcloned into blunted BamHI and SalI sites, downstream of EGFP into pLenti CMV-GFP-Puro (658–5) vector (Addgene), giving rise to pLenti148-spongeA/B. miR-148b depleted 4175-TGL were generated via lentiviral infection as described in Orso, Quirico et al 2016 [22]. For transient transfections, Anti-miR^™^ Inhibitors (AM 17000), including a scrambled miRNA negative control (AM 17100) (Invitrogen Life Technologies), were used. MDA-MB-231 cells were seeded in 6-well plates at 50% of confluency and immediately transfected using HiPerFect Transfection Reagent (QIAGEN, Stanford, CA), anti-miR and Opti-Mem I medium, as instructed. The cells were incubated at 37 °C in a CO2 incubator for 24 h, and the medium was replaced with fresh normal growth medium.

### RNA extraction, reverse transcription, and real-time PCR

Total RNA was extracted from tumor cells using Trizol reagent (Invitrogen Life Technologies). miRNA were reverse transcribed using TaqMan^®^ microRNA Reverse transcription kit (Applied Biosystems, Foster City, CA) from 10 ng total RNA according to the manufacturer’s instructions. For mRNA detection, 1 μg of DNase-treated RNA (DNA-free kit; Ambion, Austin, TX) was retrotranscribed with RETROscript reagents (Ambion), and qPCRs were performed with gene-specific primers. Quantitative normalization was performed on the expression of the housekeeping U6 for miRNAs or Actin for mRNAs. The relative expression levels between samples were calculated using the comparative delta CT (threshold cycle number) method (2^−ΔΔ*C*T^) with a control sample as reference point.

### miRNA profiling (TaqMan Low-Density Array (TLDA)

TLDA human miRNA panel is a quantitative qPCR that can simultaneously evaluate the expression levels of up to 365 different miRNAs on a single card. Briefly, 50 ng of miRNAs were converted into specific cDNAs and subsequently quantified using the TaqMan MicroRNA TLDA card, containing 365 human TaqMan miRNA sequences, and including RU6B, RNU4, and RNU44 as endogenous controls. miRNA relative expression was normalized against endogenous controls and untreated breast miRNAs according to the following threshold cycle (CT) calculation: 2−ΔCT, where ΔCT = (CT miRNA − CT miRNAs endogenous). To find consistently differentially expressed miRNAs after melatonin treatment, the data were subjected to analysis. miRNAs showing at least 1.5-fold regulation were considered to be differentially expressed.

### Protein extraction and Western Blotting

To evaluate protein expression, cells were plated and treated with melatonin in a dose of 1 mM during 48 h. At the end of the treatment, total proteins were extracted using a boiling buffer containing 0.125 mol/L Tris/HCl, pH 6.8, and 2.5% sodium dodecyl sulphate (SDS) and heated for 5 min at 95°C. Proteins were separated electrophoretically (25 or 50 μg) by SDS polyacrylamide gel electrophoresis (PAGE) and electroblotted onto Nitrocellulose membrane (BioRad^®^, Milano, Italy). Membranes were blocked in 5% blotto nonfat milk (Santa Cruz Biotechnology, Santa Cruz, CA) and Phosphate Buffered Saline PBS-Tween buffer (137 mM NaCl, 2.7 mM KCl, 8 mM Na_2_HPO_4_, 1.46 mM KH_2_PO_4_, 0.1% Tween-20) for one hour. The membranes were incubated with appropriate primary antibody for c-Myc at 1:500 (Santa Cruz) and HSP-90 at 1:2000 (Santa Cruz) overnight at 4°C. Then the membrane was incubated with PBS-Tween buffer for 1 h at room temperature and visualized by enhanced chemiluminescence “Clarity Western ECL" (Bio Rad^®^) for visualization on the "Chemidoc Touch Image Fusion" (Bio Rad^®^) apparatus. Finally, quantification was performed with the aid of image analyzer software Image J.

### Statistical analyses

The results have been previously submitted to a descriptive analysis to determine the normal range. Data are presented as mean ± Standard Error of Mean (SEM) and two tailed Student’s t test was used for comparison, with * p<0.05; ** p<0.001; *** p<0.0001 considered to be statistically significant. ns. indicates a non statistically significant p-value. We used the software Image Lab for analysis of membrane protein, and GraphPad Prism4 for statistics.

## Results

### Melatonin decreases proliferation of MCF-7 and MDA-MB-231 cells

**Fig 1:**
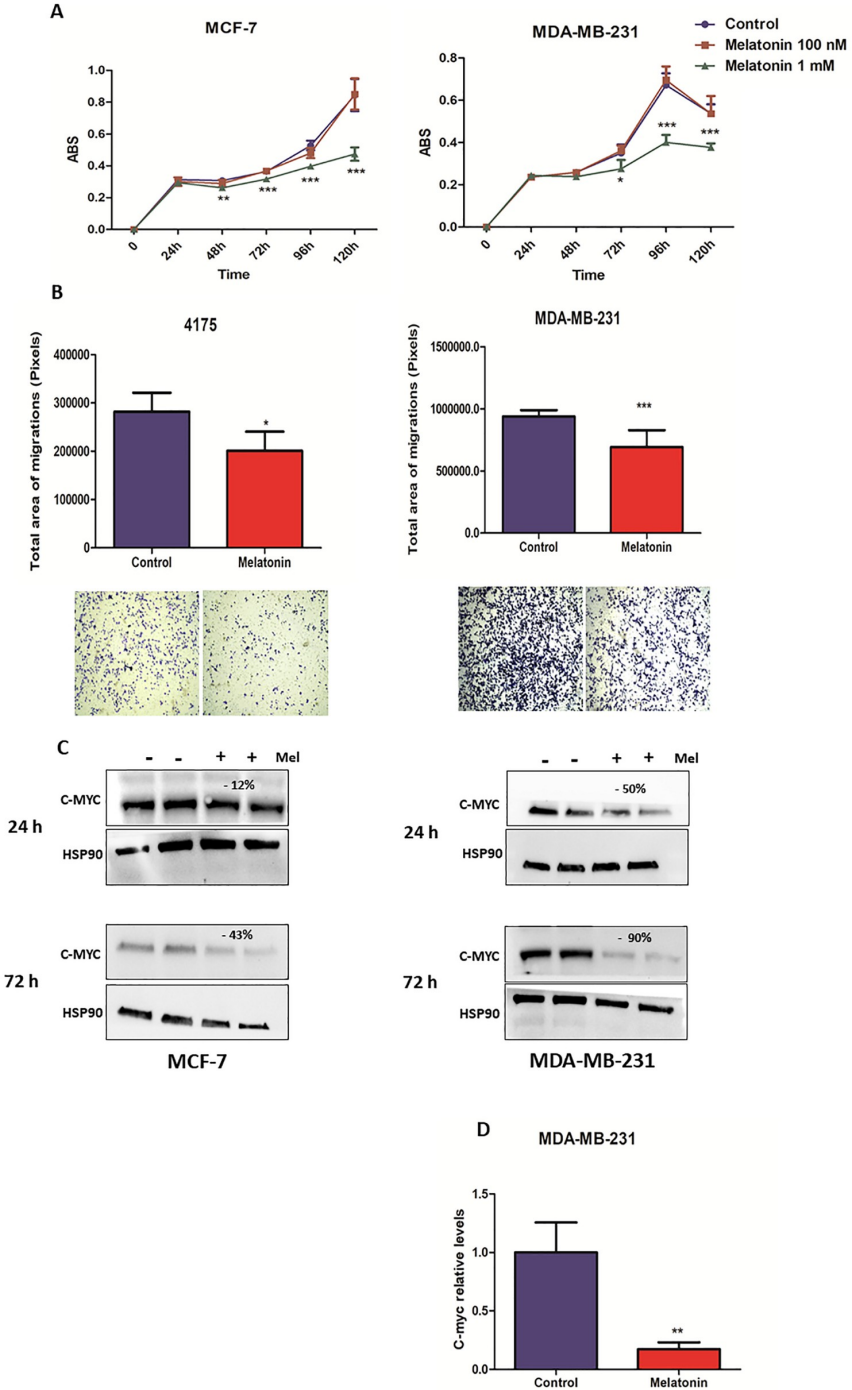
Melatonin inhibits cell proliferation, impairs migration and reduces c-Myc expression of breast cancer cells. **(A)** MCF-7 and MDA-MB-231 cells have been treated with melatonin (1 mM) for 24, 48, 72, 96 and 120 h. Each point in the graph corresponds to the mean ± SEM of the triplicates plus or minus melatonin. The proliferation ratio was measured by optical density (* p <0.05, ***p < 0.0001 treatment versus control). **(B)** MDA-MB-231 and 4175-TGL cells have been treated with melatonin for 48 h and subjected to transwell migration assays. Histograms and photographs representing cell migration rate. Data represents the mean ± SEM of the transwell area covered by migrated cells, performed in triplicates plus or minus melatonin (* p <0.05, ***p < 0.0001 treatment versus control). **(C)** MCF-7 and MDA-MB-231 cells were treated with melatonin for 24 and 72 h and protein extracts were subjected to Western Blotting with the indicated antibodies. Protein expression was quantified by ImageJ program and calculated relative to controls, normalized with the endogenous HSP90 and expressed as percentages (%). **(D)** MDA-MB-231 cells were treated for 48 h, and mRNA levels measured by qRT–PCR analyses. Results are shown as fold changes (mean±s.e.m.) relative to control cells, normalized on B-actin level. (p** < 0.001 treatment versus control). SEM = Standard Error of Mean; ABS = Absorbance.

In order to verify the antiproliferative effect of melatonin, MDA-MB-231 and MCF-7 cells were treated with 100 nM or 1 mM of melatonin for 24, 48, 72, 96 and 120 h. For this study, MCF-7 cell line was selected to be used as positive control for antiproliferative experiments, including c-Myc expression. Melatonin anticancer activities are exerted mainly binding on the estrogen receptors MT1 and MT2 and exerts its antiproliferative effects. Both ER-positive and ER-negative cell lines express the MT1 receptor; however, ER-positive tumors have an increased expression of MT1 compared to triple-receptor-negative tumors such as MDA-MB-231. The results showed a statistically significant inhibitory effect on proliferation was observed only following melatonin treatment (1 mM) at 48 h for Estrogen Receptor (ER)-positive MCF-7 cell line and at 72 h for ER-negative MDA-MB-231 cell line (Fig 1A).

### Melatonin impairs migration capability and regulates c-Myc expression on breast cancer cells

To investigate the antimetastatic effect of melatonin, transwell migration assays were performed in the presence or in the absence of this hormone on MDA-MB-231 and 4175-TGL cells. MCF-7 cells were not used in this experiment, due their poor migration capacity. As presented in Fig 1B, all cell lines treated with 1 mM melatonin showed significantly lower migration ability when compared with control cells, suggesting an anti-migratory effect of melatonin, on triple-negative breast cancer cells.

Alteration of c-Myc expression following melatonin treatment was evaluated by Western Blot analysis. Considering MCF-7 as a positive control, we also evaluated c-Myc expression on this cell line. As a result, melatonin did not modulate c-Myc in 24 h of treatment. However, in long-term treatment, melatonin led to a slight decrease of c-Myc expression. Melatonin reduced c-Myc expression on MDA-MB-231 cells after 24 h of treatment, moreover, long-term treatment with melatonin led to a strong c-Myc expression reduction (Fig 1C). Given that melatonin modulation of c-Myc was more consistent in MDA-MB-231 cells, we asked whether its effects could be transcriptional. For this reason, we evaluated c-Myc mRNA levels. A significant downregulation of c-Myc was found in cells treated with melatonin, confirming the results obtained by protein analysis and suggesting a potential transcriptional regulation of c-Myc by melatonin in triple-negative cell line (Fig 1D).

### miRNAs modulated by melatonin

In order to understand the molecular mechanism of melatonin action, we investigated whether miRNAs could be involved in these processes. Therefore, we compared the expression of 365 miRNAs in MDA-MB-231 cells treated or untreated with melatonin using “TaqMan MicroRNA Low-Density Array (TLDA)” cards in duplicate. In addition, we evaluated miR-146a and miR-148b expression by single Taqman assays. Following these analyses, melatonin treatment led to the modulation of 17 miRNAs, 11 upregulated and 6 downregulated. Among these miRNAs, 5 upregulated miRNAs (miR-let-7a, miR-let-7c, miR-10a, miR-210 and miR-148b, see Table 1) were selected for further analyses and validations.

**Table 1:** List of melatonin-regulated miRNAs in MDA-MB-231 cells.

**Down-regulated miRNAs** < 0.5-fold down-regulated miRNA	Hsa-miR-518
	Hsa-miR-520f
	Hsa-miR-576
	Hsa-miR-545
	Hsa-miR-200c
	Hsa-miR-548d
**Up-regulated miRNAs**> 1.5-fold up-regulated miRNA	Hsa-miR-10a
	Hsa-miR-452
	Hsa-miR-let-7a
	Hsa-miR-425
	Hsa-miR-17
	Hsa-miR-296
	Hsa-miR-182b
	Hsa-miR-210
	Hsa-miR-let-7c
	Hsa-miR-10b
	Has-miR-148b

We performed qRT-PCR analysis to validate the results obtained from the arrays and from independent investigations. miRNAs were considered significantly altered only when they exhibited a mean fold-change >1.5 relative to the controls, a *p* value < 0.05. Furthermore, we investigated the role of melatonin on miRNA modulation in another breast cancer cell line, MCF-7. Consistent with the previous analyses, qRT-PCR data demonstrated that out of 5 deregulated miRNAs, only miR-148b and miR-210 were significantly modulated by melatonin in MDA-MB-231 cells. Conversely, melatonin could not affect miR-148b and miR-210 in MCF-7 cells (Table 2).

**Table 2:** miR-148b and miR-210 are modulated by melatonin (1 mM) on MDA-MB-231 breast cancer cells. Results are shown as fold changes (mean±s.e.m.) relative to control cells, normalized on U6 level. (p * < 0.05, treatment versus control). SEM = Standard Error of Mean.

miRNAs	MDA-MB-231 Fold change	MCF-7 Fold change	Timepoints
**miR-let-7a**	0.91	0.69	24h
	1.25	0.75	72h
**miR-let-7c**	1.05	1.01	24h
	1.48	0.48	72h
**miR-10a**	1.03	---	24h
	0.94		72h
**miR-10b**	1.14	---	24h
	1.72		72h
**miR-24**	1.17	0.71	24h
	1.08	0.86	72h
**miR-100**	0.83	---	24h
	0.77		72h
**miR-146a**	1.18	---	24h
	0.91		72h
**miR-148b**	1.53*	1.10	24h
	1.41	1.28	72h
**miR-210**	1.65*	0.90	24h
	1.81	0.66	72h

### Is there a functional link between deregulated miRNAs and melatonin efficacy?

Considering that miR-148b and miR-210 were up-regulated by melatonin in MDA-MB-231 cells, we assessed the involvement of these miRNAs in the negative modulation of c-Myc, and the ability of melatonin to reduce cell migration. Therefore, in order to identify whether miR-148b or miR-210 could mediate melatonin effects, MDA-MB-231 or 4175-TGL cells were engineered for a stable downregulation of miR-148bsponges to obtain miR-148b depleted cells or transiently transfected with miRNA-210 inhibitor.

**Fig 2:**
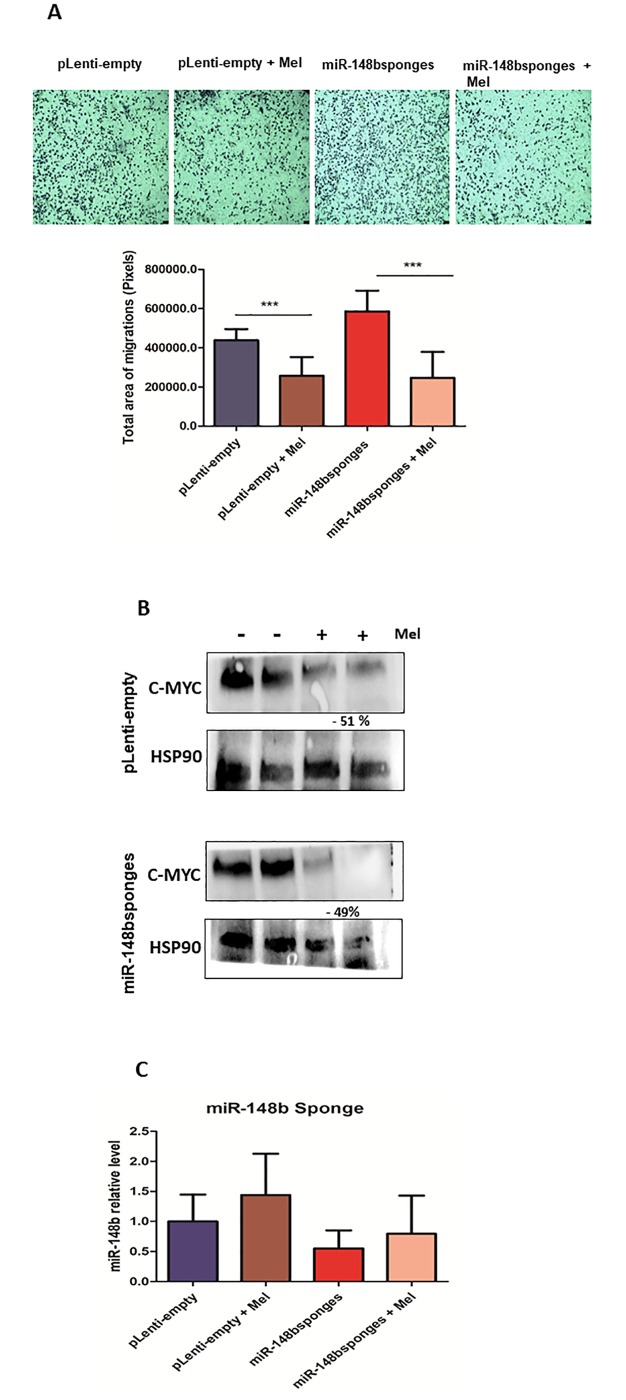
Melatonin reduces migration and c-Myc expression independently of miR-148b (A) 4175-TGL cells transduced with miR-148bsponges or pLenti-empty lentivirus have been treated with melatonin (1 mM) for 48 h and subjected to transwell migration assays. Histograms represent cell migration rate. Data represents the mean ± SEM of the transwell area covered by migrated cells, performed in triplicates plus or minus melatonin (p*** < 0.0001 treatment versus control). **(B)** 4175-TGL cells transduced as described above were treated with melatonin for 48 h and protein extracts were subjected to Western Blotting with the indicated antibodies. Protein expression was quantified by ImageJ program and calculated relative to controls, normalized with the endogenous HSP90 and expressed as percentages (%). **(C)**. 4175-TGL cells transduced as described above were treated for 48 h and miRNA levels measured by qRT–PCR analyses. Results are shown as fold changes (mean±s.e.m.) relative to control cells, normalized on U6 level. (p > 0.05 treatment versus control). SEM = Standard Error of Mean.

Cells were transduced or transfected with an empty control (pLenti-empty) or a scrambled anti-miRNA (Neg. Control) and were used for comparisons. All cells were then treated, or not, with melatonin. Transwell assays were used to evaluate migration. Melatonin significantly affected cell migration even in miR-148b depleted cells (Fig 2A), suggesting that melatonin-dependent migration reduction is miR-148b independent. In addition, a modulation of c-Myc was evaluated by Western Blot analysis. Melatonin reduced c-Myc expression in 4175-TGL cells previously transduced with miR-148bsponges or pLenti-empty. However, there was no difference in c-Myc expression between empty and miR-148b depleted cells. Thus, downregulation of c-Myc expression seems to be melatonin-dependent but miR-148b-independent (Fig 2B). Melatonin-induced miR-148 expression was blocked when cells expressed miR-148bsponges (Fig 2C). Based on c-Myc and migration data we can postulate that increased levels of miR-148b are an epiphenomenon induced by melatonin but independent on its action.

**Fig 3:**
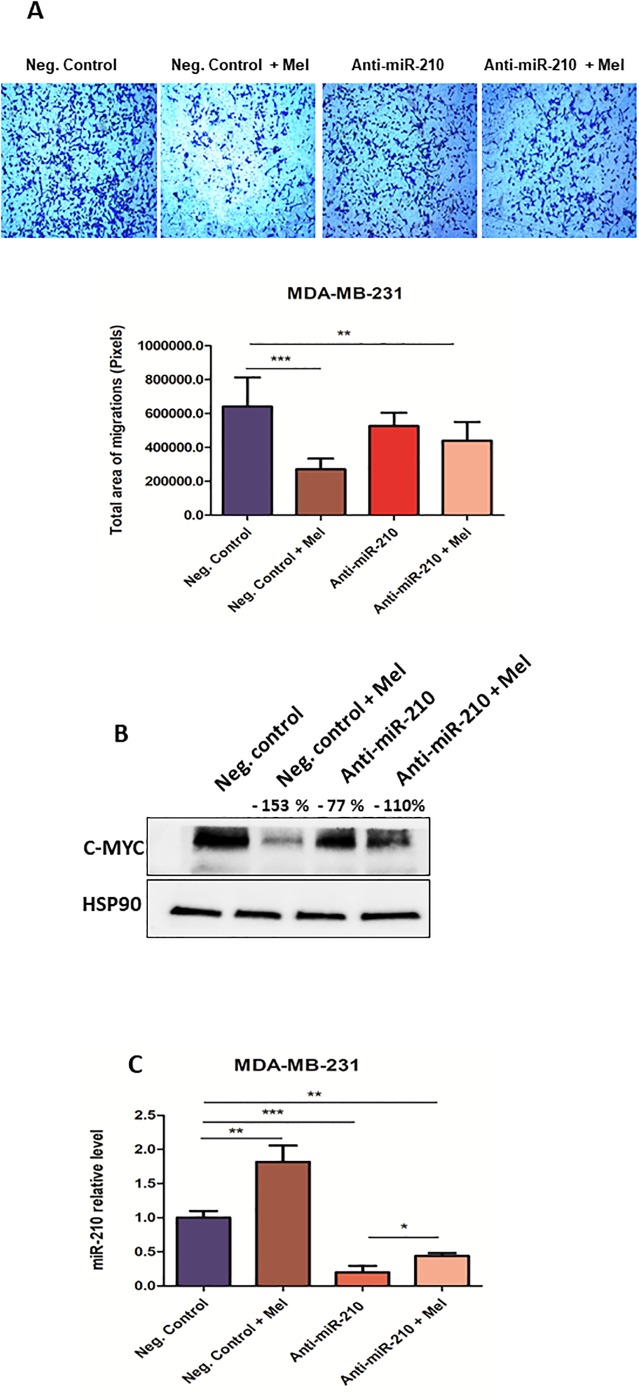
Melatonin reduces migration and c-Myc expression independently of miR-210. **(A)** MDA-MB-231 cells transiently transfected with anti-miR-210 have been treated with melatonin (1 mM) for 48 h and subjected to transwell migration assays. Histograms represent cell migration rate. Data represent the mean ± SEM of the transwell area covered by migrated cells, performed in triplicates plus or minus melatonin (**p < 0.001, p*** < 0.0001 treatment versus control) **(B)** MDA-MB-231 cells transfected as described above were treated with melatonin for 48 h and protein extracts were subjected to Western Blotting with the indicated antibodies. Protein expression was quantified by ImageJ program and calculated relative to controls, normalized with the endogenous HSP90 and expressed as percentages (%) **(C)** MDA-MB-231 cells transfected as described above were treated with melatonin for 48 h and miRNA levels measured by qRT–PCR analyses. Results are shown as fold changes (mean±s.e.m.) relative to control cells, normalized on U6 level. (* p < 0.05, **p < 0.001, ***p < 0.0001 treatment versus control). SEM = Standard Error of Mean.

Regular and low miR-210 expressing MDA-MB-231 cells were treated, or not, with melatonin and miR-210 levels were verified. Regarding migration, decreased cell movement was observed for melatonin treated cells, and a moderate decrease was observed in untreated cells with low levels of miR-210 when compared to controls (Fig 3A). When c-Myc expression was evaluated, decreased c-Myc was also found when miR-210 levels were reduced, suggesting that melatonin-induced c-Myc decrease was independent from the observed miR-210 up-regulation (Fig 3B and 3C).

## Discussion

Melatonin has been considered an important natural antitumor agent, and high levels of this hormone negatively correlates with the risk of developing many types of cancers [23,24]. Our functional studies revealed a role for melatonin in migration inhibition in MDA-MB-231 triple-negative cell line. In breast cancer, melatonin`s antitumor actions have been previously evaluated especially in the ER-positive tumors, where melatonin acts antiestrogenically through its membrane receptors, called MT1 and MT2 [25–28]. On this line, considering that melatonin anticancer activities are exerted mainly binding on the estrogen receptors, we considered MCF-7 cells as a positive control for melatonin treatment. Consistent with the literature, in our findings regarding ER-positive cells, melatonin induced an inhibitory effect on cell proliferation, which is statistically significant after 48 h. More interestingly, on triple-negative MDA-MB-231 cells, melatonin was also able to impair proliferation. However, a longer time following melatonin treatment was required. Based on these findings, we can suggest that melatonin`s effectiveness requires long periods on triple-negative cells. Similarly, a recent study of Kim and Cho (2017) showed that melatonin (10 μM) reduced cell migration and invasion of triple-negative breast cancer cell line MDA-MB-231. However, the authors revealed no effect on proliferation after melatonin treatment [29]. In the present study, only the highest concentration (1 mM) was effective in metastatic and ERa-negative cells. To achieve therapeutic effects, endogenous substances are commonly administered in much higher concentrations than those corresponding to the physiological concentration. The range of melatonin in human serum is assumed as physiological 10^−9^ M and pharmacological 10^−6^ M or higher. The detected concentration of melatonin in plasma at night is approximately 0.1 nM– 1 nM corresponding to peak nighttime, among 2 AM to 5 AM, and 10 pM corresponding to day time serum values in humans [15,30,31,32].

It has been established that melatonin binds to membrane receptors MT1 and MT2 and exerts its antiproliferative effects [18]. Both ER-positive and ER-negative cell lines express the MT1 receptor; however, relevant studies have shown that ER-positive tumors have an increased expression of MT1 compared to triple-receptor-negative tumors such as MDA-MB-231 line [25,32]. Since MDA-MB-231 cells present a molecular deficiency in MT1 signaling pathway, melatonin could also influence ER-negative cells via processes that do not involve the MT1/MT2 membrane receptors. The lipophilic nature of melatonin can allow its passage through the cell membrane and its interactions with a number of transcription factors and nuclear binding sites that are involved in modulation of breast cancer cell proliferation [27,32,33].

Considering that miR-148b and miR-210 were up-regulated by melatonin, we assessed the relation of these miRNAs in the negative modulation of c-Myc. In order to investigate the link of these miRNAs, we looked for genes related to cell proliferation and metastasis. Relevantly, we observed c-Myc modulation after melatonin treatment in MCF-7 and MDA-MB-231 cell lines. Interestingly, melatonin reduced c-Myc expression on MDA-MB-231 cells after 24 h of treatment. However long-term treatment with melatonin led to a strong reduction of c-Myc expression. In contrast, melatonin does not modulate c-Myc expression after 24 h on MCF-7 cells. A significant reduction was observed after just 72 h. To further explore the metastatic-suppressive actions of melatonin, we performed transwell migration assay using MDA-MB-231 and 4175-TGL (a highly metastatic cell line derived from MDA-MB-231 cell line). As expected, we found that melatonin was able to reduce cell migration in both triple-negative breast cancer cell lines.

We have previously shown that melatonin can reduce HIF-1a and VEGF gene and protein expression[34,35]. Nevertheless, there is no evidence so far about underlying mechanism between melatonin and oncostatic effects. We can speculate that one possible evidence would be via microRNAs. miRNAs are small non-coding RNA molecules responsible for modulating several encoding genes [36,37]. Currently, there are several studies presenting evidence that miRNAs play a crucial role in tumor progression. Therefore, it is essential to understand how miRNAs intervene in melatonin`s effects on tumor cell proliferation and migration. So far, the mechanism remains obscure. Accordingly, for the first time, the present study identified a set of miRNAs differentially modulated in melatonin-treated triple-negative MDA-MB-231 cells.

Among the mostly differentially expressed miRNAs, miR-210 and miR-148b were found upregulated following melatonin treatment and chosen for functional investigations. miR-148b has been investigated in several tumor types, acting as a tumor suppressor. Its low expression is closely related to an increase in both tumor progression and the number of metastases [38]. According to Zhang et al. (2015) high expression of miR-148b in human hepatocarcinoma cells (HepG2) leads to a decrease in c-Myc protein levels. Instead, in our study increased miR-148b does not influence melatonin`s effect on c-Myc or migration in a positive or negative manner in breast cancer cells [39].

miR-210 is considered an oncogenic miRNA, that exhibits HIF-1α, Von-Hippel Lindau tumor suppressor (VHL) mediated regulation. HIF-1α promotes increased expression of miR-210, and its miRNA promotes the stabilization of HIF-1α, suggesting a positive feedback loop [40]. According to the literature, high levels of miR-210 leads to an increased cancer progression, reduced cell cycle arrest and enhanced cancer migration in MCF-7 cells [41]. Zhang et al. (2009) revealed that high levels of miR-210 in human colon carcinoma cells HCT116 led to increased c-Myc expression through the reduction of MNT, a known MYC antagonist, responsible to impair proproliferative and proapoptotic functions of c-Myc [42]. Conversely, here we show that miR-210 is upregulated by melatonin in tumor cells. However, a positive miR-210 modulation by melatonin in these cells shows no increase on cell proliferation or migration. Many studies suggest that miR-210 can also act as a tumor suppressor, inhibiting tumor initiation. In agreement, Giannakakis et al. (2008) demonstrated that induction of miR-210 down-regulates E2F3, an important transcription factor involved in the regulation of the cell cycle in ovarian cancer cells.[43] In addition, melatonin negatively alters c-Myc expression and impairs migration on cells showing reduced levels of miR-210.

Similarly, as miR-148b results revealed, melatonin has modulatory action on both miRNAs, however this modulation is not directly associated with the decrease of c-Myc and cell migration. Moreover, treatments of tumor cells with melatonin can lead to miRNA modulations, which are not necessarily causative. Regarding c-Myc modulation, a possible hypothesis could be due to this gene not being directly associated as a direct target for miR-148b and miR-210 in triple-negative breast tumor cells. In addition, melatonin may act to decrease c-Myc through negative modulation of other genes, such as HIF-1a. According to a recent study published by Vriend and Reiter (2016), since melatonin plays an important role as an antioxidant, its action leads to a decrease in ROS (Reactive Oxygen Species) and regulation of several genes, such as HIF-1 and VEGF, exhibiting an indirect inhibitory function and independent ER action of this hormone [28,44,45].

According to Reiter et al. (2017) the basic role of melatonin at the molecular level remains unclear. Widely beneficial functions of melatonin are being identified and considered only epiphenomena from some melatonin’s fundamental function [32]. Regarding melatonin-induced miRNAs modulation, we speculate it could be just “epiphenomenon” due to general dicer/drosha deregulations by this hormone. Similarly, in a study of Mori et al. (2016) melatonin (1 uM) was able to downregulate miR-24 expression through inhibition of hnRNP A1, a protein involved in both mRNA splicing and miRNA maturation [46]. More importantly, the authors observed that mRNA levels were not regulated by melatonin, suggesting that melatonin has a post-transcriptional regulation on hnRNP A1. On this line, the study of Mori et al. (2016) is in accordance with our findings, suggesting possible miRNA modulation by melatonin in an indirect way. On the other hand, it is also possible that melatonin acts via the subtle modulations of groups of miRNAs and not a specific one, which renders the study of miRNA intervention difficult to perform. It is already known that one miRNA can potentially modulate the expression of hundreds of genes, which makes comprehensive prediction of its effect on the cell very complex.

In this way, further studies must be developed to link melatonin to miRNAs, c-Myc, cell proliferation and migration. The present study was important to understand the role of melatonin in a possible metastatic pathway involving the regulation of miRNAs on MDA-MB-231 triple-negative cell line. Accordingly, these results must be strengthened, and further experiments are being performed with the objective of elucidating the specific action of melatonin in this process.

## Supporting information

S1 Fig (DOCX)

S1 Table (DOCX)

S1 Data (PDF)

S2 Data (PDF)

S3 Data (PDF)

S4 Data (XLSX)

S5 Data (PDF)

S6 Data (PDF)
