# Supplementary material for: The role of melatonin on miRNAs modulation in triple-negative breast cancer cells
Source: PLoS One. 2020 Feb 3;15(2):e0228062. doi: 10.1371/journal.pone.0228062 (PMC6996834; doi:10.1371/journal.pone.0228062)

**Mycoplasma testing results**

- MDA-MB-231 cell line
- 4175 cell line


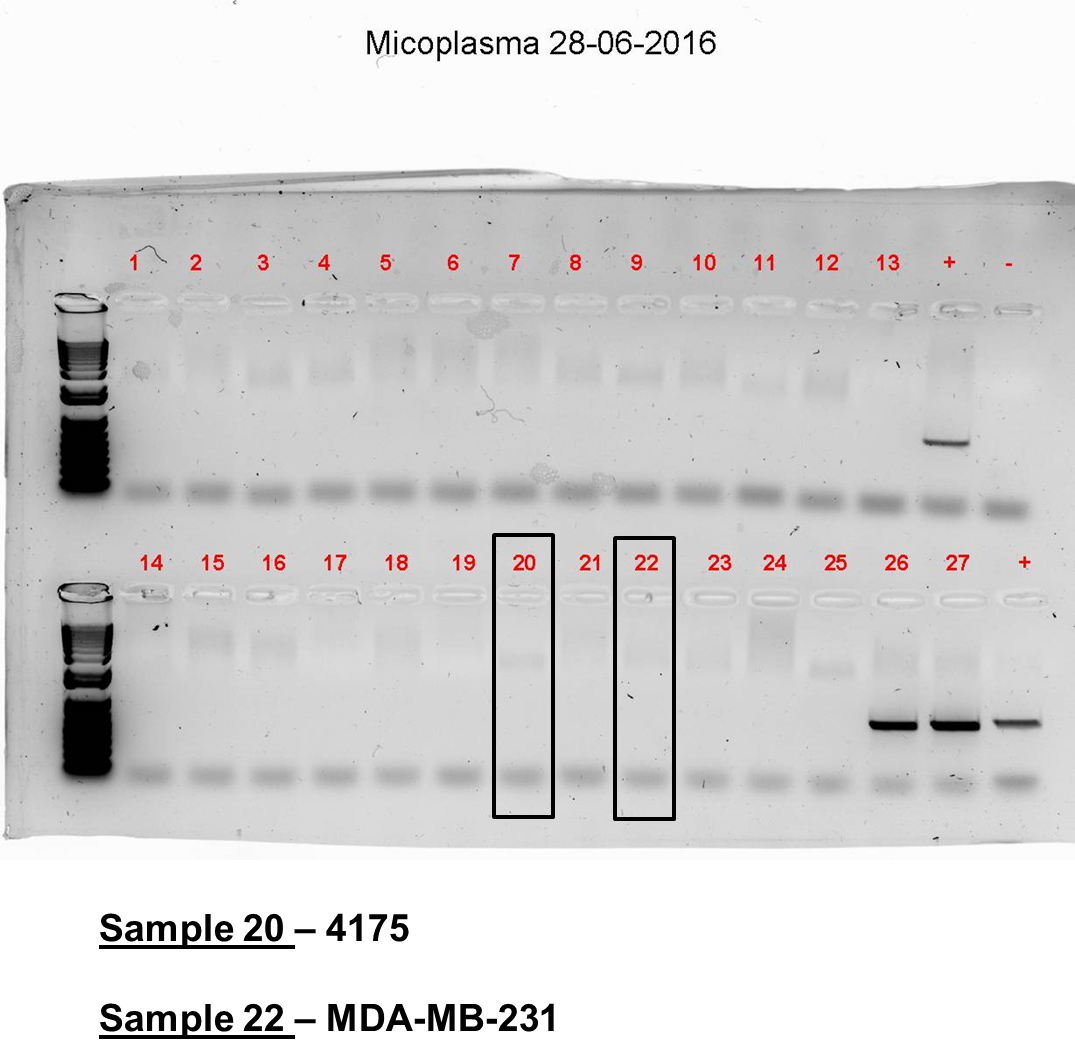


**Mycoplasma testing results**

- MCF-7 cell line


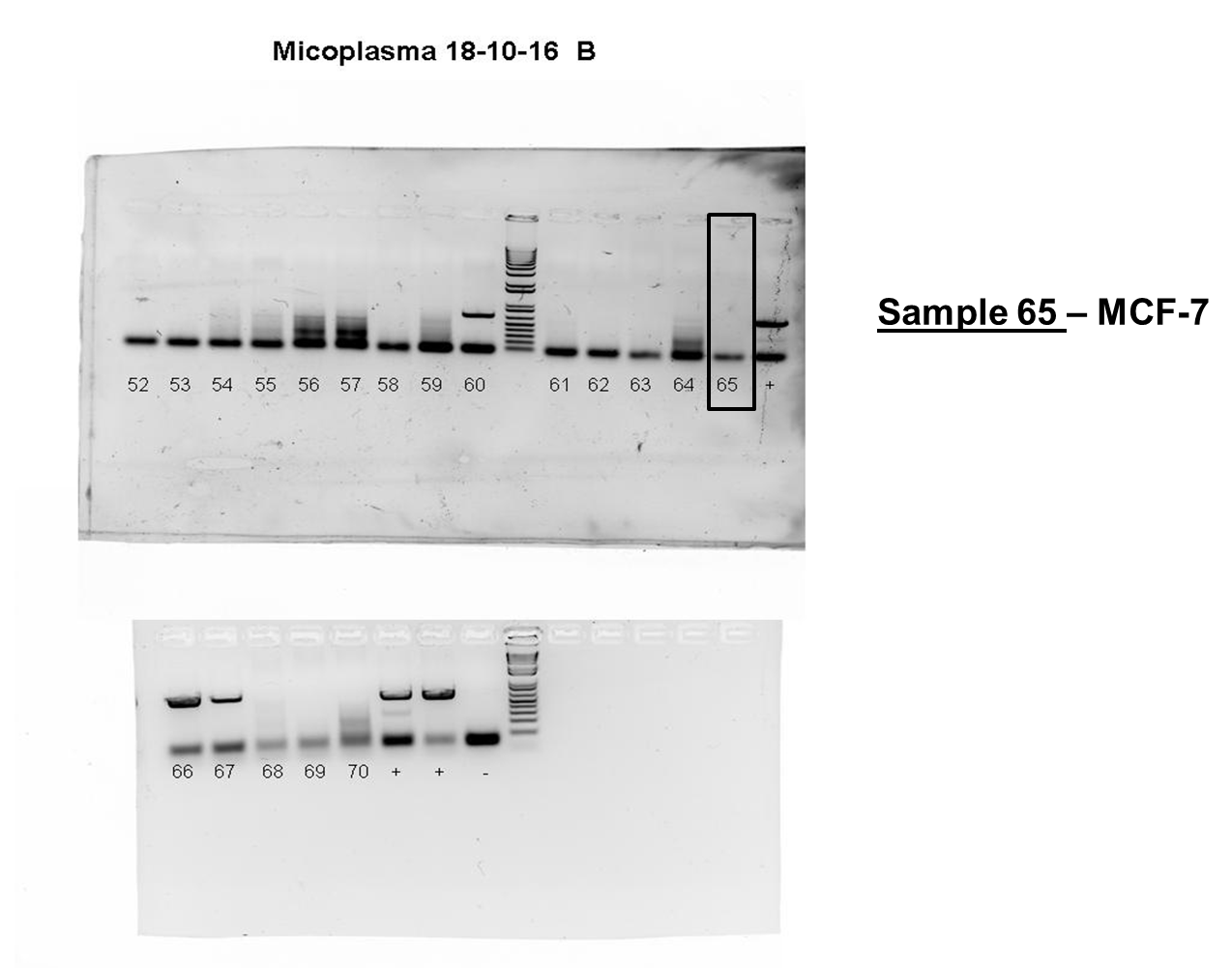

Supplement: S1 Fig — (DOCX) [file pone.0228062.s001.docx]
