# Supplementary figures and images for: The role of melatonin on miRNAs modulation in triple-negative breast cancer cells
Source: PLoS One. 2020 Feb 3;15(2):e0228062. doi: 10.1371/journal.pone.0228062 (PMC6996834; doi:10.1371/journal.pone.0228062)

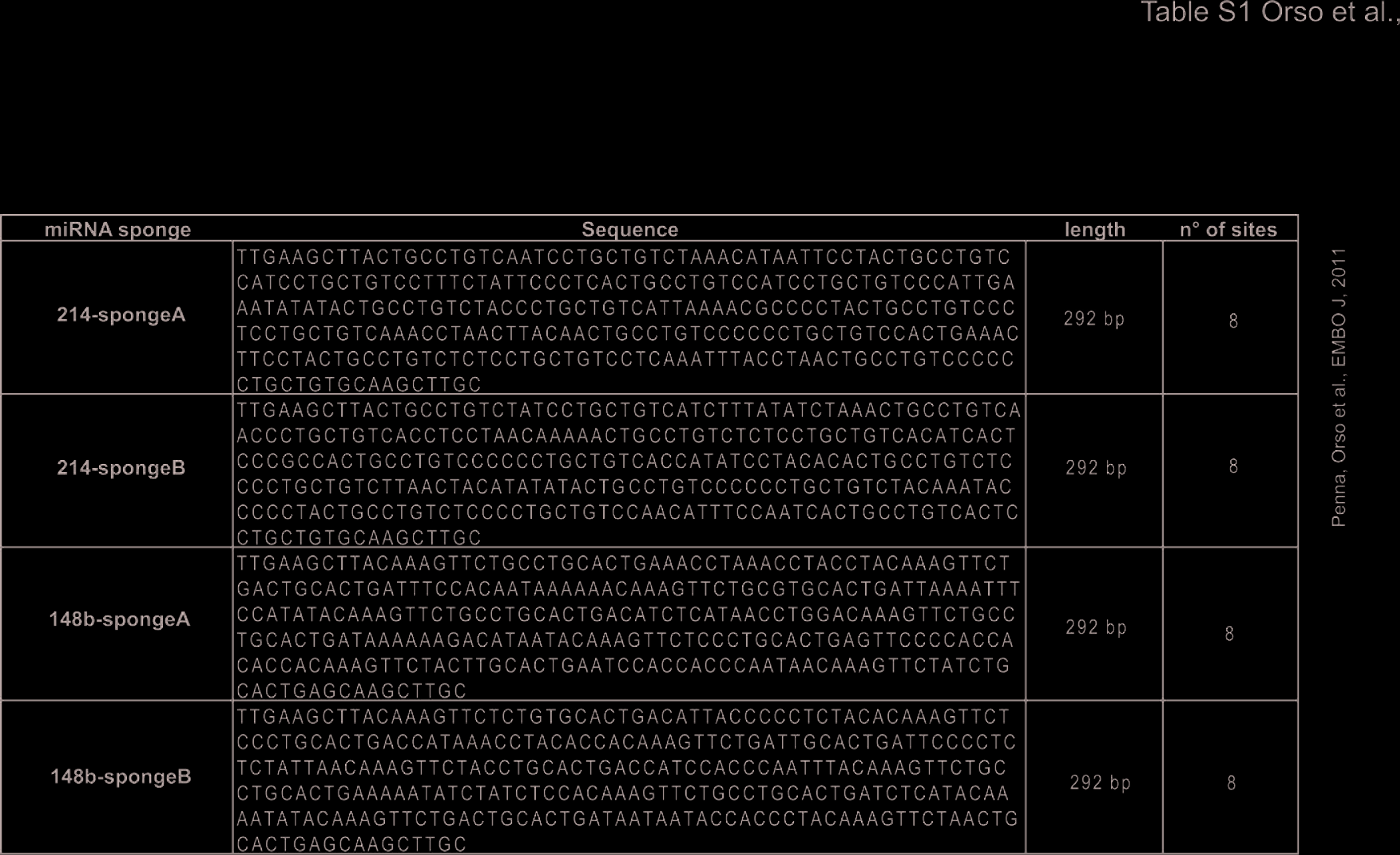

Supplement: S1 Table — (DOCX) [file pone.0228062.s002.docx]
