# Supplementary material for: The role of melatonin on miRNAs modulation in triple-negative breast cancer cells
Source: PLoS One. 2020 Feb 3;15(2):e0228062. doi: 10.1371/journal.pone.0228062 (PMC6996834; doi:10.1371/journal.pone.0228062)
Supplement: S1 Data — (PDF) [file pone.0228062.s003.pdf]

MDA-MB-231					
**Slice**	**Count**	**Total Area**	**Average Size**	**%Area**	**Mean**
Control MDA-MB-231.	707	876616	1239,909	27,086	255
Control MDA-MB-231.	664	904180	1361,717	27,938	255
Control MDA-MB-231.	664	930644	1401,572	28,756	255
Control MDA-MB-231.	677	893623	1319,975	27,612	255
Control MDA-MB-231.	715	907435	1269,14	28,038	255
Control MDA-MB-231.	657	960132	1461,388	29,667	255
Control MDA-MB-231.	645	944385	1464,163	29,18	255
Control MDA-MB-231.	645	1000472	1551,119	30,913	255
Control MDA-MB-231.	727	925164	1272,578	28,586	255
Control MDA-MB-231.	627	1042835	1663,214	32,222	255
		**938548,6**			
		**51049,98735**			
Melatonin MDA-MB-231.	524	1024591	1955,326	31,658	255
Melatonin MDA-MB-231.	888	550604	620,05	17,013	255
Melatonin MDA-MB-231.	677	694260	1025,495	21,452	255
Melatonin MDA-MB-231.	614	778353	1267,676	24,05	255
Melatonin MDA-MB-231.	766	735503	960,187	22,726	255
Melatonin MDA-MB-231.	708	694730	981,257	21,466	255
Melatonin MDA-MB-231.	646	609569	943,605	18,835	255
Melatonin MDA-MB-231.	578	602182	1041,837	18,607	255
Melatonin MDA-MB-231.	712	613317	861,4	18,951	255
Melatonin MDA-MB-231.	476	617754	1297,803	19,088	255
		**692086,3**			
		**136029,8438**			

4175					
**Slice**	**Count**	**Total Area**	**Average Size**	**%Area**	**Mean**
Control 4175.	456	154458	338,724	4,773	255
Control 4175.	465	147819	317,89	4,567	255
Control 4175.	510	235366	461,502	7,272	255
Control 4175.	612	273634	447,114	8,455	255
Control 4175.	456	191532	420,026	5,918	255
Control 4175.	670	482479	720,118	14,908	255
Control 4175.	560	366322	654,146	11,319	255
Control 4175.	657	430761	655,648	13,31	255
Control 4175.	615	339302	551,711	10,484	255
Control 4175.	522	197897	379,113	6,115	255
		**281957**			
		**117610,1663**			
Melatonin 4175.	384	186729	486,273	5,77	255
Melatonin 4175.	323	126506	391,659	3,909	255
Melatonin 4175.	438	206963	472,518	6,395	255
Melatonin 4175.	440	200169	454,93	6,185	255
Melatonin 4175.	528	287669	544,828	8,889	255
Melatonin 4175.	465	224044	481,815	6,923	255

Melatonin 4175.	435	197404	453,802	6,099	255
Melatonin 4175.	489	204530	418,262	6,32	255
Melatonin 4175.	447	169682	379,602	5,243	255
Melatonin 4175.	470	179875	382,713	5,558	255
Melatonin 4175.	460	241886	525,839	7,474	255
		**202314,2727**			
		**39239,29654**			
