# Supplementary material for: The role of melatonin on miRNAs modulation in triple-negative breast cancer cells
Source: PLoS One. 2020 Feb 3;15(2):e0228062. doi: 10.1371/journal.pone.0228062 (PMC6996834; doi:10.1371/journal.pone.0228062)
Supplement: S2 Data — (PDF) [file pone.0228062.s004.pdf]

MDA-MB-231

Time 24 h						Media	Desvpad
Control	0,252532	0,230391	0,223905	0,23988	0,235794	0,2365	0,010781
Mel 100 nM	0,250184	0,238283	0,234518	0,232997	0,227995	0,236795	0,008345
Mel 1 mM	0,244185	0,233262	0,248321	0,240942	0,254642	0,24427	0,008004

Time 48 h							
Control	0,279489	0,254982	0,246818	0,256852	0,25697	0,259022	0,012176
Mel 100 nM	0,2677	0,251051	0,252773	0,244487	0,27827	0,258856	0,01378
Mel 1 mM	0,24307	0,235257	0,244276	0,238152	0,230342	0,238219	0,005724

Time 72 h							
Control	0,320931	0,373272	0,404963	0,347099	0,305134	0,35028	0,040089
Mel 100 nM	0,354903	0,399299	0,343669	0,369571	0,342321	0,361952	0,02357
Mel 1 mM	0,273473	0,346117	0,267967	0,254431	0,237759	0,275949	0,041578

Time 96 h							
Control	0,654295	0,697524	0,581095	0,710089	0,714567	0,671514	0,05588
Mel 100 nM	0,748301	0,658195	0,651949	0,642613	0,781641	0,69654	0,063812
Mel 1 mM	0,439575	0,368676	0,424932	0,412159	0,358538	0,400776	0,035472

Time 120 h							
Control	0,557794	0,496993	0,554987	0,488547	0,590147	0,537693	0,043377
Mel 100 nM	0,615877	0,511503	0,448793	0,476473	0,633968	0,537323	0,083243
Mel 1 mM	0,356601	0,393396	0,393585	0,355704	0,385726	0,377002	0,019298

MCF-7

Time 24 h						Media	Desvpad
Control	0,297978	0,340411	0,303666	0,30664	0,322115	0,314162	0,017182
Mel 100 nM	0,345945	0,276896	0,303383	0,294921	0,284558	0,301141	0,02699
Mel 1 mM	0,314422	0,311542	0,278224	0,28755	0,283046	0,294957	0,016813

Time 48 h							
Control	0,320938	0,307574	0,320303	0,286111	0,304779	0,307941	0,014216
Mel 100 nM	0,274141	0,327356	0,27078	0,291616	0,284148	0,289608	0,022652
Mel 1 mM	0,268456	0,255496	0,252496	0,261336	0,270632	0,261683	0,007886

Time 72 h							
Control	0,378799	0,344521	0,350261	0,370702	0,379445	0,364746	0,016339
Mel 100 nM	0,378237	0,371771	0,377108	0,358003	0,353278	0,367679	0,011381
Mel 1 mM	0,321358	0,330311	0,309911	0,33524	0,290207	0,317405	0,018001

Time 96 h							
Control	0,488483	0,521883	0,513411	0,554295	0,562806	0,528176	0,030473
Mel 100 nM	0,468925	0,435001	0,512973	0,506463	0,478914	0,480455	0,031365
Mel 1 mM	0,411792	0,381019	0,416584	0,393635	0,382699	0,397146	0,016382

Time 120 h							
Control	0,821675	0,846892	0,813841	1,007182	0,731121	0,844142	0,100978
Mel 100 nM	0,864908	0,802889	1,015571	0,801718	0,772613	0,85154	0,097677
Mel 1 mM	0,435078	0,487404	0,506652	0,517062	0,424881	0,474215	0,041915
