# Supplementary material for: The role of melatonin on miRNAs modulation in triple-negative breast cancer cells
Source: PLoS One. 2020 Feb 3;15(2):e0228062. doi: 10.1371/journal.pone.0228062 (PMC6996834; doi:10.1371/journal.pone.0228062)
Supplement: S3 Data — (PDF) [file pone.0228062.s005.pdf]

MDA-MB-231 - 24 hs

c-myc

Sample	mean	mean -blank	average	ratio		
Control	16086,685	13556,435	10972,5295	3,73705364	1	**100**
Control	10918,874	8388,624				
Mel	9726,118	7195,868	6343,338	1,89816639	0,50793127	**50,79313**
Mel	8021,058	5490,808				
background	2530,25					

HSP90

Sample	mean	mean -blank	average
Control	4660,977	3041,502	2936,1445
Control	4450,262	2830,787	
Mel	4936,814	3317,339	3341,824
Mel	4985,784	3366,309	
background	1619,475		

MDA-MB-231 - 72 hrs

c-myc

Sample	mean	mean -blank	average	ratio		
Control	4391,593	3056,635	3237,275	0,61440814	1	**100**
Control	4752,873	3417,915				
Mel	1545,277	210,319	213,35	0,06198523	0,10088608	**10,08861**
Mel	1551,339	216,381				
background	1334,958					

HSP90

Sample	mean	mean -blank	average
Control	9271,63	5125,431	5268,9325
Control	9558,633	5412,434	
Mel	7223,483	3077,284	3441,949
Mel	7952,813	3806,614	
background	4146,199		

MCF-7 - 24 hrs

C-myc

Sample	mean	mean -blank	average	ratio		
Control	14029,912	9269,625	9711,513	2,5743958	1	**100**
Control	14913,688	10153,401				
Mel	15778,402	11018,115	10468,486	2,24977488	0,87390404	**87,3904**

Mel	14679,144	9918,857
background	4760,287	

HSP90

Sample	mean	mean -blank	average
Control	5497,217	1570,233	3772,3465
Control	9901,444	5974,46	
Mel	9108,92	5181,936	4653,126
Mel	8051,3	4124,316	
background	3926,984		

MCF-7 - 72 h

C-myc

Sample	mean	mean -blank	average	ratio		
Control	8785,304	6783,212	6615,903	1,65810588	1	**100**
Control	8450,686	6448,594				
Mel	6262,831	4260,739	3790,877	0,94905671	0,57237401	**57,2374**
Mel	5323,107	3321,015				
background	2002,092					

HSP90

Sample	mean	mean -blank	average
Control	5457,161	4221,025	3990,0365
Control	4995,184	3759,048	
Mel	5098,464	3862,328	3994,363
Mel	5362,534	4126,398	
background	1236,136		

Anti-miR-210

c-myc

Sample	mean	mean -blank	ratio		
Neg. Control	7109,275	5158,233	2638,933	1	**100**
Neg. Control + Mel	2892,325	941,283	-1261,316	-0,47796439	**-47,796439**
Anti-miR-210	4881,182	2930,14	620,252	0,23503893	**23,5038934**
Anti-miR-210 + Mel	4420,295	2469,253	-303,804	-0,1151238	**-11,51238**
background	1951,042				

HSP90

Sample	mean	mean -blank
Neg. Control	3363,601	2519,3
Neg. Control + Mel	3046,9	2202,599
Anti-miR-210	3154,189	2309,888

Anti-miR-210 + Mel	3617,358	2773,057
background	844,301	

pLent-Empty

c-myc

Sample	mean	mean -blank	average	ratio		
Control	10273,656	7681,179	6371,7705	1,70104579	1	**100**
Control	7654,839	5062,362				
Mel	5439,927	2847,45	2672,1975	0,82741075	0,48641298	**48,6413**
Mel	5089,422	2496,945				
background	2592,477					

HSP90

Sample	mean	mean -blank	average
Control	13912,608	4830,458	3745,796
Control	11743,284	2661,134	
Mel	12902,609	3820,459	3229,59
Mel	11720,871	2638,721	
background	9082,15		

miR-148b sponge

Sample	mean	mean -blank	average	ratio		
Control	8347,757	5354,685	5600,081	2,32690846	1	**100**
Control	8838,549	5845,477				
Mel	4725,903	1732,831	1732,831	1,18369969	0,50870058	**50,87006**
Mel	--					
background	2993,072					

HSP90

Sample	mean	mean -blank	average
Control	8089,007	2810,405	2406,6615
Control	7281,52	2002,918	
Mel	7083,457	1804,855	1463,911
Mel	6401,569	1122,967	
background	5278,602		
