# Supplementary material for: The role of melatonin on miRNAs modulation in triple-negative breast cancer cells
Source: PLoS One. 2020 Feb 3;15(2):e0228062. doi: 10.1371/journal.pone.0228062 (PMC6996834; doi:10.1371/journal.pone.0228062)
Supplement: S5 Data — (PDF) [file pone.0228062.s007.pdf]

BMR Genomics srl

Rev. 1,1
13/05/2015

Certificato di analisi

Prenotazione di riferimento Nr. 128272 | Data di emissione 29/10/2015

Alla c.a. Della Dr.ssa Fiorella Balzac
Login: torino
Ente: Università degli Studi di Torino
e-mail: fiorella.balzac@unito.it
Telefono: 0116706419

Fasi dell'Analisi

Fase1: Amplificazione
Amplificazione del materiale inviato mediante il kit PowerPlex Fusion System secondo le specifiche del produttore.
La reazione di amplificazione è stata effettuata su termociclatore Eppendorf Mastercycler.

Fase2: Elettroforesi ed analisi
Corsa elettroforetica su sequenziatore capillare “Applied Biosystems 3130XL”:
– Fascio da 16 capillari (36cm)
– Polimero di corsa POP7
– Software di gestione strumento: Data Collection Software Ver. 3.0
– Software di analisi: Genemapper ID Ver. 3.2.1

Risultati dell'analisi

L'esito della corsa elettroforetica sui 24 marcatori genetici del kit PowerPlex Fusion System ha prodotto i seguenti profili genetici:

Marcatori	A837-4175n	A838-ma2	A839-t47d	A840-hbl100
**AMEL**	X	X	X	X
**D3S1358**	16	15-17	15-17	14-16
**D1S1656**	15-17	16-17.3	15-16	12-16.3
**D2S441**	14-15	11	14	11
**D10S1248**	16	15-16	17	13
**D13S317**	13	11-14	12	12
**Penta E**	11	10-12	7-14	7
**D16S539**	12	9	10	9-12
**D18S51**	16	12-17	17	16
**D2S1338**	20-21	16-24	24	18-24
**CSF1PO**	12-13	11-12	11-13	10
**Penta D**	11-14	9-15	10-12	12
**TH01**	7-9.3	8	6	6-8
**vWA**	15-18	16-17	14	16
**D21S11**	30-33.2	29-30	28-31	28
**D7S820**	8	9	11	8-12
**D5S818**	12	12	12	11-12
**TPOX**	8-9	8-10	11	8
**DYS391**	-	-	-	-
**D8S1179**	13	11-14	13	12-15
**D12S391**	17-18	18-21	19	18

**D19S433**	11-14	13-14.2	14	15
**FGA**	22-23	20-23	23	25 V
**D22S1045**	16	16	15	15-16

Nomenclatura:

(n): picchi deboli o stutter particolarmente intensi

Operatore che ha effettuato l'analisi

Giorgia Bovo

Note:
Il certificato si riferisce ai risultati ottenuti dai campioni forniti dall'utente, costituiti da DNA genomici, sottoposti ad analisi.

AZIENDA CON SISTEMA DI GESTIONE QUALITÀ CERTIFICATO DA DNV GL = ISO 9001 =
