# Supplementary material for: The role of melatonin on miRNAs modulation in triple-negative breast cancer cells
Source: PLoS One. 2020 Feb 3;15(2):e0228062. doi: 10.1371/journal.pone.0228062 (PMC6996834; doi:10.1371/journal.pone.0228062)
Supplement: S6 Data — (PDF) [file pone.0228062.s008.pdf]

Alla c.a. Francesca Orso, PhD
Molecular Biotechnology Center Dept.
Oncological Sciences
University of Turin Via Nizza, 52
10126 Torino
Italy
login: torino
Phone: +39 011 670 6448
Fax: +39 011 670 6432
e-mail: francesca.orso@unito.it

Fasi dell'Analisi

Fase1: Amplificazione
Amplificazione del materiale inviato mediante il kit Cell ID(tm) System secondo le specifiche del produttore.
La reazione di amplificazione è stata effettuata su termociclatore Applied Byosistems "GeneAmp PCR System 9700".

Fase2: Elettroforesi ed analisi
Corsa elettroforetica su sequenziatore capillare "Applied biosystems 3130XL":
- Fascio da 16 capillari (36cm)
- Polimero di corsa POP7
- Software di gestione strumento: Data Collection Software Ver. 3.0
- Software di analisi: Genemapper ID Ver. 3.2.1

Risultati dell'analisi

L'esito della corsa elettroforetica sui 10 marcatori genetici del kit Cell ID(tm) System ha prodotto i seguenti profili genetici:

Nomenclatura:

(n) = Picco debole o picco stutter molto intenso

Difficile Interpretazione

Marcatore	792A-MDAMB231	
TH01	7-9.3	√
D21S11	33.2	
D5S818	12	√
D13S317	13	√
D7S820	8-9	√
D16S539	12	√
CSF1PO	12-13	√
AMEL	X	√
vWA	15-18	√
TPOX	8-9	√

Note e Commenti:

Operatore che ha effettuato l'analisi

Giorgio Malacrida

Note:

Il certificato si riferisce ai risultati ottenuti dai campioni forniti dall'utente, costituiti da DNA genomici, sottoposti ad analisi.
